# California Case Study of Wildfires and Prescribed Burns: PM_2.5_ Emissions, Concentrations, and Implications for Human Health

**DOI:** 10.1021/acs.est.3c06421

**Published:** 2024-03-14

**Authors:** Laura Kiely, Soroush E. Neyestani, Samiha Binte-Shahid, Robert A. York, William C. Porter, Kelley C. Barsanti

**Affiliations:** † Chemical and Environmental Engineering, University of California Riverside, Riverside, California 92521, United States; ‡ Now at: Scion, Christchurch 8011, New Zealand; § Department of Environmental Sciences, University of California Riverside, Riverside, California 92521, United States; ∥ Department of Environmental Science, Policy, and Management, University of California Berkeley, Berkeley, California 94720, United States; ⊥ Atmospheric Chemistry Observations and Modeling, U.S. National Science Foundation National Center for Atmospheric Research, Boulder, Colorado 80301, United States

**Keywords:** wildfires, prescribed burns, air quality, CMAQ, smoke, PM_2.5_

## Abstract

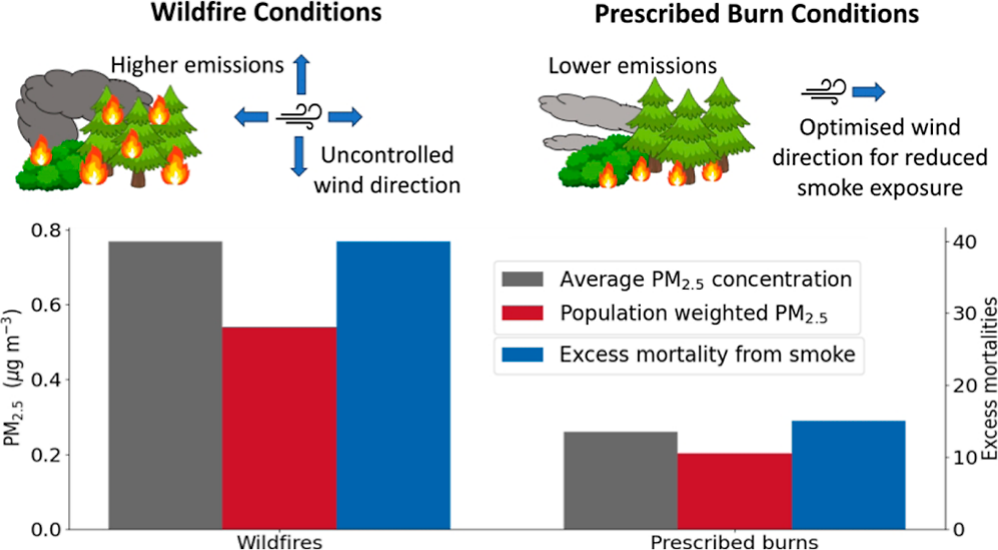

Wildfires are a significant threat to human health, in part through degraded air quality. Prescribed burning can reduce wildfire severity but can also lead to an increase in air pollution. The complexities of fires and atmospheric processes lead to uncertainties when predicting the air quality impacts of fire and make it difficult to fully assess the costs and benefits of an expansion of prescribed fire. By modeling differences in emissions, surface conditions, and meteorology between wildfire and prescribed burns, we present a novel comparison of the air quality impacts of these fire types under specific scenarios. One wildfire and two prescribed burn scenarios were considered, with one prescribed burn scenario optimized for potential smoke exposure. We found that PM_2.5_ emissions were reduced by 52%, from 0.27 to 0.14 Tg, when fires burned under prescribed burn conditions, considerably reducing PM_2.5_ concentrations. Excess short-term mortality from PM_2.5_ exposure was 40 deaths for fires under wildfire conditions and 39 and 15 deaths for fires under the default and optimized prescribed burn scenarios, respectively. Our findings suggest prescribed burns, particularly when planned during conditions that minimize smoke exposure, could be a net benefit for the impacts of wildfires on air quality and health.

## Abstract 2

Prescribed burning could be effective in reducing health impacts associated with wildfires due to exposure to PM_2.5_ concentrations, particularly when smoke transport is considered in planning prescribed burns.

## Introduction

Human-fire interactions have a long history in the western US. Historically, fire was used by native populations as a vegetation management tool, in which frequent controlled fires served to assist hunting, promote desired vegetation growth, and prevent wildfires.^1^ Starting in the late 1800s, fire suppression became a key component of forest policy, leading to the current high fuel buildup in the western US.^2^ Due to this buildup of fuels as well as changes in climate, catastrophic wildfires (defined by damage to natural and built environments and the endangerment of people) are increasing in frequency–a trend which is expected to continue.^3−5^ Prescribed burning is the practice of using controlled and low-intensity burns, when conditions are favorable, to reduce understory fuel loads while leaving the majority of the overstory undamaged.^2^ Reducing fuel loads thus reduces the likelihood of high-intensity wildfires.^6^ To restore ecological function and influence wildfire behavior at the landscape scale, applications of prescribed fire need to increase substantially.^7^

Large wildfires emit significant amounts of fine particulate matter (PM_2.5_), resulting in worsened regional air quality and adverse health impacts.^8−10^ Northern California is heavily forested, and fires in the region can have greater PM emissions than elsewhere in the US due to the high surface fuel loads and live biomass that is consumed during a crown fire.^9^ Because prescribed fires can lower future wildfire severity and fuel consumption, prescribed burning may be considered a net benefit in the context of the air quality impacts of wildfires in Northern California and similarly forested areas. While prescribed burns also emit PM_2.5_ and negatively impact air quality,^11,12^ those impacts may not be as severe as wildfires due to differences in fuel conditions, fuel consumption, emissions, and seasonal meteorological patterns.^10,13,14^ For example, prescribed burns are generally limited to spring and fall, while wildfires, driven by available fuel, mostly occur during the summer months.^15^ The seasonal differences in typical weather conditions between the two fire types lead to important differences in fuel conditions and the transport of emitted pollutants.

The air quality impacts of prescribed burns need to be compared with those of wildfires when prescribed burning is evaluated as a tool to mitigate catastrophic wildfires. Making a comprehensive evaluation is complex and is hindered by gaps in current understanding and modeling capabilities. In a review of literature on fire in the US, Jaffe et al.^9^ highlighted the need for a better understanding of the differences between wildfire and prescribed burn emissions and how burn strategies could minimize air quality impacts. Altshuler^16^ and Williamson et al.^14^ highlighted the need to model the differences in both emissions and transport when comparing the air quality impacts of prescribed burns and wildfires. One of the challenges in quantitatively comparing the air quality impacts of wildfires and prescribed burns is that studies tend to focus on specific fire events, which vary in size and location, and often occur in different regions of the US.^12^

To overcome some of the limitations of prior studies, here, we have considered fires hypothetically occurring in the same locations burning under wildfire and prescribed burn conditions to better quantify the relative air quality impacts and allow for a direct comparison. This novel approach bridges the gap between studies showing how prescribed burning can reduce emissions^6,13^ and studies showing the health impacts of fire smoke.^10,11^ Modeling the emissions of wildfires and prescribed burns requires knowledge of fire-specific fuel consumption, combustion efficiency, and emission factors, which are likely different between fire types. This information is becoming increasingly available through new literature and fire modeling frameworks.^10,17^ We have taken historical wildfires from 2012 and modeled them both as they actually occurred and under hypothetical scenarios using meteorological and fuel conditions suitable for prescribed burning. We have evaluated how burn conditions and seasons can impact fire emissions and transport and what this means for the air quality and health impacts of fires. Finally, we have considered two prescribed burn scenarios with burning occurring on different days to show the range of impacts and highlight the importance of burn timing, particularly in the context of wind direction and smoke exposure.

## Methods

**Figure 1:**
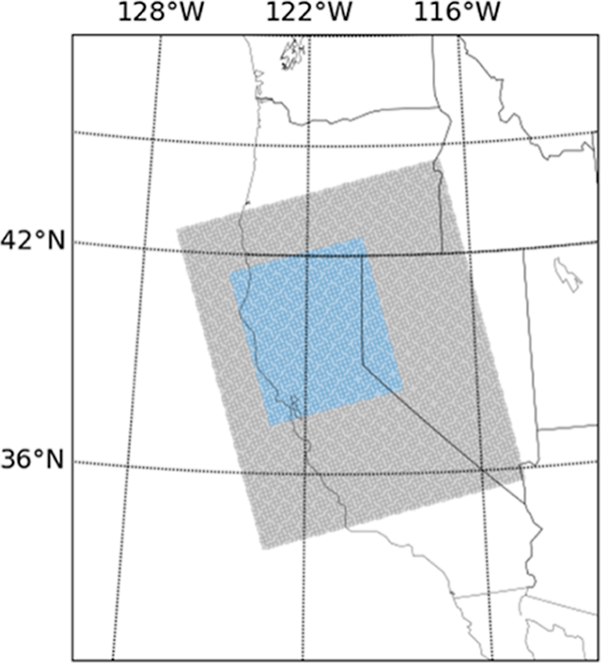
CMAQ domain (gray) and study area (blue).

Fire emissions data were set up for three fire scenarios, “wildfires”, “Rx1”, and “Rx2”, all based on 2012 wildfire data for Northern California. The modeling domain is shown in Figure 1. The wildfire scenario represents fires burning under wildfire conditions, as detected, and the two prescribed burn (Rx) scenarios represent fires with the same location and area burning under prescribed burn conditions (i.e., on days with conditions suitable for prescribed burning). Under the first prescribed burn scenario (Rx1), temperature, wind speed, relative humidity, and soil moisture were considered when determining the suitability of a day, while under the second prescribed burn scenario (Rx2), potential smoke exposure was also considered. The Environmental Protection Agency (EPA) Community Multiscale Air Quality (CMAQ)^18^ chemical transport model was used to predict PM_2.5_ concentrations, and a relative risk function was used to estimate excess short-term mortality associated with PM_2.5_.

### Fire Emissions and Scenarios

The fire emissions used in this study were created by combining burned area, fuel consumed, and emissions factors (EFs) as follows
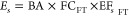
1where *E*_*s*_ is the emissions of species *s* (g), BA is the burned area (km^2^), FC_FT_ is the fuel consumed of fuel type FT (kg/km^2^), and EF_*s* FT_ is the EF (g/kg) of species *s* for fuel type FT. Theburned area was determined from the MODIS satellite product derived from surface reflectance, available at daily 500 m resolution.^19^ This product is used in the global fire emissions database (GFED) and has been used previously for fires in California.^20,21^

For the Rx1 scenario, the burned area for each fire detected by MODIS was moved to occur on days in the spring (March through May) or fall (September through November), corresponding to conditions typically considered suitable for prescribed burning. These seasonal windows represent the common times during which fuel moisture, meteorology, air quality, and permitting align to allow prescribed fires to take place.^22^ We used environmental conditions representative of those under which prescribed burns typically occur in forests: 20 feet above-ground wind speed <5.36 m/s (12 mph), temperature <29.5 °C (85 °F), relative humidity between 0.25 and 0.45, and soil moisture between 0.15 and 0.3 m^3^/m^3^. The ranges in environmental conditions allow for the fact that appropriate burn windows are also dependent on local factors, such as topography and fuel type. If multiple days had environmental conditions within the required ranges, the day with the lowest wind speed was chosen for the burning to occur, which is consistent with what would be done during a prescribed burn to minimize escape risk. If, for any fire location, there were no days with environmental conditions within these ranges, days with wind speed <15 mph and relative humidity up to 0.6 were considered when finding a new day for that fire. The expanded ranges, needed for only 2% of the total burned area, are not unreasonable for prescribed burns, particularly in locations where, due to weather conditions, pile burning is done as an alternative to broadcast burning. For the Rx2 scenario, days on which fires would lead to high-population-weighted PM_2.5_ exposure were removed before the same environmental filters were applied as for Rx1. Selected “no burn” periods were April 27–May 2, May 28–June 2, October 26–28, and November 2–5 (see Supporting Information for further details on how these periods were chosen). Large wildfire areas which burned over consecutive days in the wildfire scenario could be burned on a single day in the Rx scenarios if the entire area was within one grid cell for the environmental conditions.

The environmental data for all simulations was from the Modern-Era Retrospective Analysis for the Research and Applications Meteorological Data Product (MERRA-2) at 0.5 × 0.65° resolution.^23^ While the prescribed burn conditions apply to the surface (∼12 m elevation), the MERRA surface layer height is up to ∼60 m in the modeled region. Wind speed at the surface is likely less than that for the surface layer of the model, particularly under trees; therefore, the wind speed criterion used for the Rx scenarios is likely conservative.

Fuel consumption was calculated using the CONSUME model,^24^ which takes fuel loading from the fuel characteristic classification system (FCCS),^25^ available at 30 m resolution. The CONSUME model requires fuel moisture, which was estimated from soil moisture content using MERRA-2 and the BlueSky modeling framework literature,^17^ as described in the Supporting Information. For the Rx scenarios, because fires occur on different days than for the wildfire scenario, there are different fuel moistures associated with the fires. Canopy consumption, a key difference between wildfires and prescribed burns,^26^ was set at 50% for wildfires and 0% for prescribed burns, as recommended in BlueSky. For all scenarios, the percentage of shrub blackened was set to 50%, as recommended in BlueSky. CONSUME may overestimate fuel consumption for some fuels in prescribed burns,^27^ meaning that the emissions reduction between wildfires and prescribed burns estimated in this study may be conservative. The CONSUME model has been used previously to calculate fuel consumption for wildfires and prescribed burns in Northern California.^28,29^

The EFs were taken from Urbanski (2014) as used in the first-order fire effects model (FOFEM)^31^ and are shown in Table 1 for PM_2.5_ and in Table S2 for CO and CO_2_. FOFEM includes a category specifically for Western US forests, with different EFs available for wildfires and prescribed burns. EFs are available for forest, shrubland, and grassland fuel categories, with separate EFs for woody and duff smoldering. Each of the different FCCS fuel beds within the burned area was assigned to one of these fuel categories using the percentages of fuel loading coming from canopy, shrub, nonwoody, or woody (see Supporting Information for further details). If the largest percentage was from canopy and woody fuel, the fuel bed was assigned as forest. If the largest percentage was from shrub, the fuel bed was assigned as shrubland. If the largest percentage was nonwoody, the fuel bed was assigned as grassland. In each of these fuel categories, there was some fraction of woody fuels and duff that was burned during smoldering combustion, and the relevant EFs were applied.^30,48^

**Table 1:** Emission Factors (EF) for Different Land Cover Types. EFs Are Given in g/kg^c^

Cover type	PM_2.5_ EF
western forest—Rx STFS^a^	17.57
western forest—WF STFS^a^	23.2
shrubland STFS^a^	7.06
grassland STFS^a^	8.51
woody RSC^b^	33
duff RSC^b^	35.3

a Short-term flaming and smoldering.

b Residual smoldering combustion.

c The EFs for Western Forest fuel types are different for wildfires (WF) and prescribed burns (Rx).

### Chemical Transport Modeling

The CMAQv5.3.3 model^18^ was used to simulate PM_2.5_ concentrations across Northern California for 2012 under the wildfire and prescribed burn scenarios and a control scenario with no fire emissions. The changes in PM_2.5_ concentrations due to fires were calculated for each fire scenario as the difference between model runs with and without fires. Figure 1 shows the model domain at 12 km resolution on a lambert conformal grid. Vertically resolved concentration profiles distributed with CMAQ and reflective of a marine environment were used to create initial and boundary conditions. To minimize the impact of the initial and boundary conditions, a three-week spin-up period was used, and a study area at the center of the domain was selected for the air quality analysis.

In CMAQ, Carbon Bond 6 (CB06) version r3 was selected as the gas-phase chemical mechanism and AERO7 as the aerosol model, with SOA parameterized using the volatility basis set approach.^32^ SOA formation was negligible in these simulations compared with primary PM emissions. PM was represented using 3 size distributions: two Aitken modes ≤ (2.5 μm in diameter) and one accumulation mode (>2.5 μm in diameter). Anthropogenic emissions from the 2011 National Emissions Inventory were converted to model-ready inputs using the SMOKEv3.7 preprocessor through the 2011v6 platform;^33^ biogenic emissions were calculated online in CMAQ. A preprocessor for CMAQ was used to convert the fire emissions data to model-ready inputs.^34^ This included the application of a daily temporal variation for the fire emissions (Figure S2) and a vertical distribution (Figure S3). The vertical distribution was based on observed top heights of wildfire and prescribed burn plumes (around 3000 and 1300 m, respectively^35,36^). The meteorology was from the weather research and forecasting model version 3.9.1 with the Thompson scheme for microphysics,^37^ the Rapid Radiative Transfer Model for radiative transfer,^38^ the Tiedtke scheme for cumulus parameterization,^39,40^ the Mellor-Yamada-Janjic scheme for planetary boundary layer parameterization,^41^ and the Noah model for land surface physics.^42,43^ CMAQ was run offline with no feedback between the fire emissions and the meteorology. It has been found that fire emissions can impact cloud cover and cloud microphysics, affecting temperature and rainfall,^44^ which have not been considered in this study.

The CMAQ model run with wildfire emissions was evaluated using PM_2.5_ observations downloaded from the EPA.^45^ The modeled and observed daily PM_2.5_ were compared using the normalized mean biased factor (NMBF)
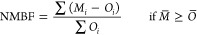
2
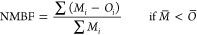
3and the normalized mean absolute error factor (NMAEF)
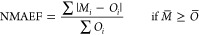
4
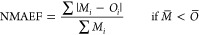
5where *M*_*i*_ and *O*_*i*_ are pairs of modeled and observed values, respectively.^46^

### Health Impacts

The impact of smoke on human health was estimated here by using exposure to increased PM_2.5_ concentrations in simulations with fires relative to the simulation without fires. Population-weighted PM_2.5_ (PW) was used to evaluate exposure, calculated as
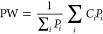
6where *P*_*i*_ is the population of grid cell *i* and *C*_*i*_ is the concentration in that grid cell. Population data were from the Gridded Population of the World (GPWv4) for 2010, available at 30 arcsecond resolution and regridded to match the model resolution. The total population in the model domain was 19.3 million.

The daily short-term excess mortality (*M*) from fires was calculated using
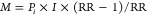
7where *P*_*i*_ is the population in grid cell *i* and *I* is the baseline mortality rate for the US in 2012 taken from the global burden of disease. The annual rate of 813 deaths per 100,000 people was converted to a daily rate per person. RR is the relative risk function
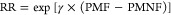
8where PMF and PMNF are the daily PM_2.5_ concentrations with and without fires, respectively, and γ is the excess mortality per unit increase in PM_2.5_. Since PM_2.5_ from fires may have a greater toxicity than PM_2.5_ from other sources,^47^ γ = 0.00101 was used to specifically represent mortality from fire-derived PM_2.5_ in the US as calculated by Chen et al.,^48^ with a 95% confidence interval of 0.001001–0.001020. This method for estimating RR and short-term mortality has been used previously for fires in the US and other countries.^49,50^

## Results

### Modeled PM_2.5_, CO, and CO_2_ Emissions

**Figure 2:**
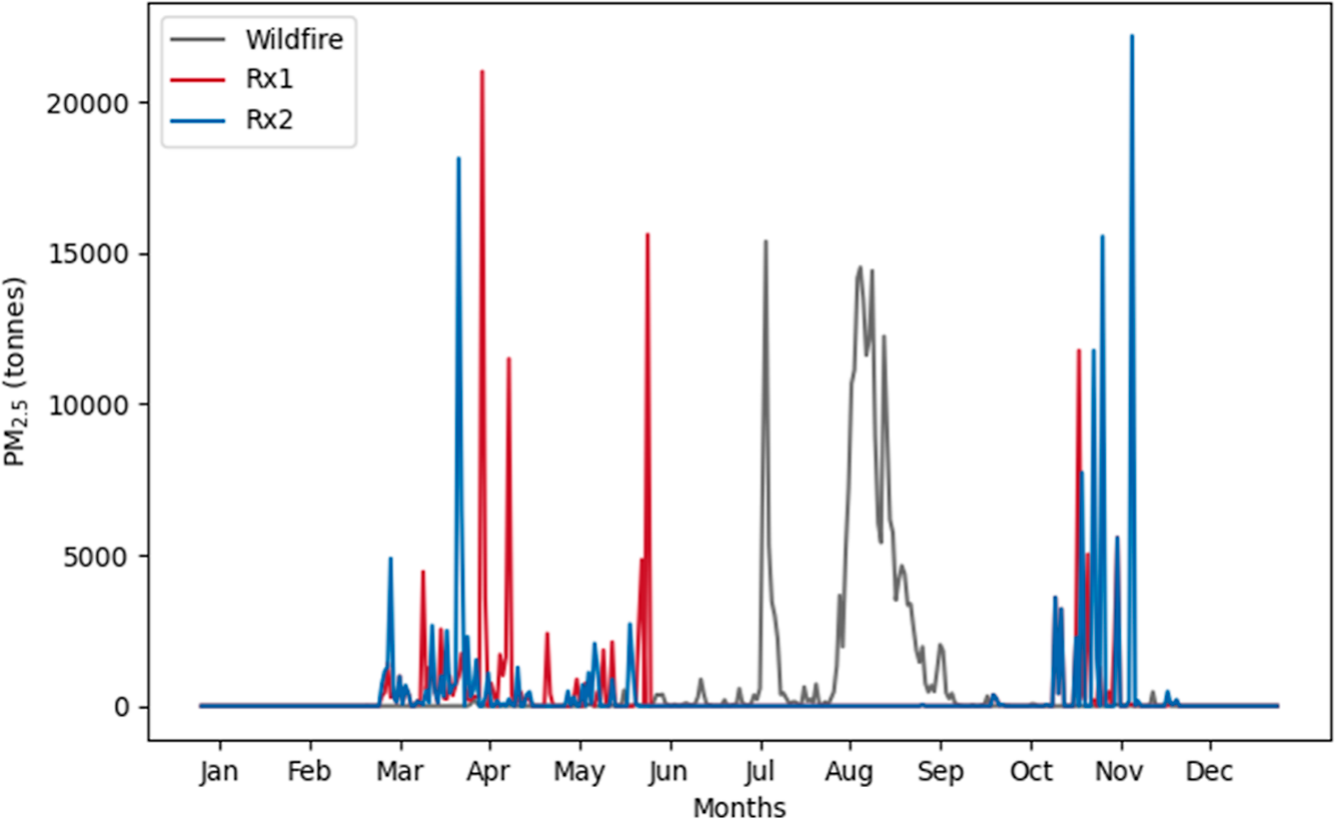
Daily total emissions of PM_2.5_ for the domain in Figure 1 under the wildfire (gray), Rx1 (red), and Rx2 (blue) scenarios.

Table 2 summarizes the burned area and the total PM_2.5_ emissions from fires in the Figure 1 domain under the wildfire and prescribed burn scenarios. CO and CO_2_ emissions can be found in Table S3. Per the study design, the fires occurred in the same locations under all scenarios, and thus the total burned area remained the same. In the domain, a total of 11,220 km^2^ burned in 2012, with 40% in tree fuels and 32% in shrub fuels. This is comparable to the burned area estimated using the Fire Inventory from NCAR (FINNv2.5) of 16,929 km^2^ and GFEDv4s of 12,024 km^2^ for the same domain. FINN uses MODIS hotspot data with a fixed burned area per fire detection,^51^ whereas MODIS burned area was used to calculate emissions in this study. The use of hotspot data likely explains the larger area burned estimated using FINN. Under the wildfire scenario 0.265 Tg of PM_2.5_ was emitted in the domain in 2012, predominantly during May to September (Figure 2). This is comparable to FINNv2.5,^52^ which resulted in an estimated 0.295 Tg of PM_2.5_ emitted, both of which are greater than the PM_2.5_ emissions estimated by GFED4s of 0.127 Tg. This difference is likely due to the lower emission factors used by GFED4s (12.9 g/kg for PM_2.5_ for temperate forests), which are intended to represent an average global temperate forest rather than a western US mixed coniferous forest. Under the Rx1 scenario, 0.138 Tg of PM_2.5_ was emitted, with 72% between March and May and 28% between October and November. Under the Rx2 scenario, 0.140 Tg of PM_2.5_ was emitted, with 46% between March and May and 54% between October and November. The minimal increase in emissions between Rx1 and Rx2 was due to differing fuel moisture conditions on the different days chosen for the burns. In addition to the decrease in the total amount of PM_2.5_ emitted in the prescribed burn scenarios relative to the wildfire scenario, the number of days with fire emissions greater than 1 tonne decreased from 194 days under wildfire conditions to 93 days under Rx1 and 80 days under Rx2. Total CO emissions were reduced from 1.73 Tg under the wildfire scenario to 0.94 and 0.96 Tg under Rx1 and Rx2, respectively, and total CO_2_ emissions were reduced from 16.25 to 9.65 and 9.76 Tg, respectively. The relative reduction in PM_2.5_ between the two scenarios was larger than that for CO or CO_2_ emissions, likely due to the relative difference in PM_2.5_ and CO_2_ EFs for wildfires and prescribed burns. For any grid cell in the domain, total annual emissions were reduced when fires burned under prescribed burn conditions compared to wildfires.

**Table 2:** Burned Area, Fuel Consumption, and Emissions of PM_2.5_ under the Wildfire and Prescribed Burn Scenarios for the Model Domain Shown in Figure 1^a^

	wildfires	Rx1	Rx2
burned area (km^2^)	11,220	11,220 (0%)	11,220 (0%)
% burned area on trees/shrub/grass	40/32/16	40/32/16	40/32/16
fuel consumption (Tg)	10.56	6.29 (40%)	6.36 (40%)
PM_2.5_ (Tg)	0.265	0.138 (48%)	0.140 (47%)

a The percentage reduction for each variable for the prescribed burn scenarios compared with the wildfire scenario is shown in brackets. The percentages of the total burned area which occurred on fuel types categorized as trees, shrubs, and grass are shown. Burned area, fuel loading, and fuel consumption split by fuel type can be found in Table S4.

### Modeled PM2.5 Concentrations

**Figure 3:**
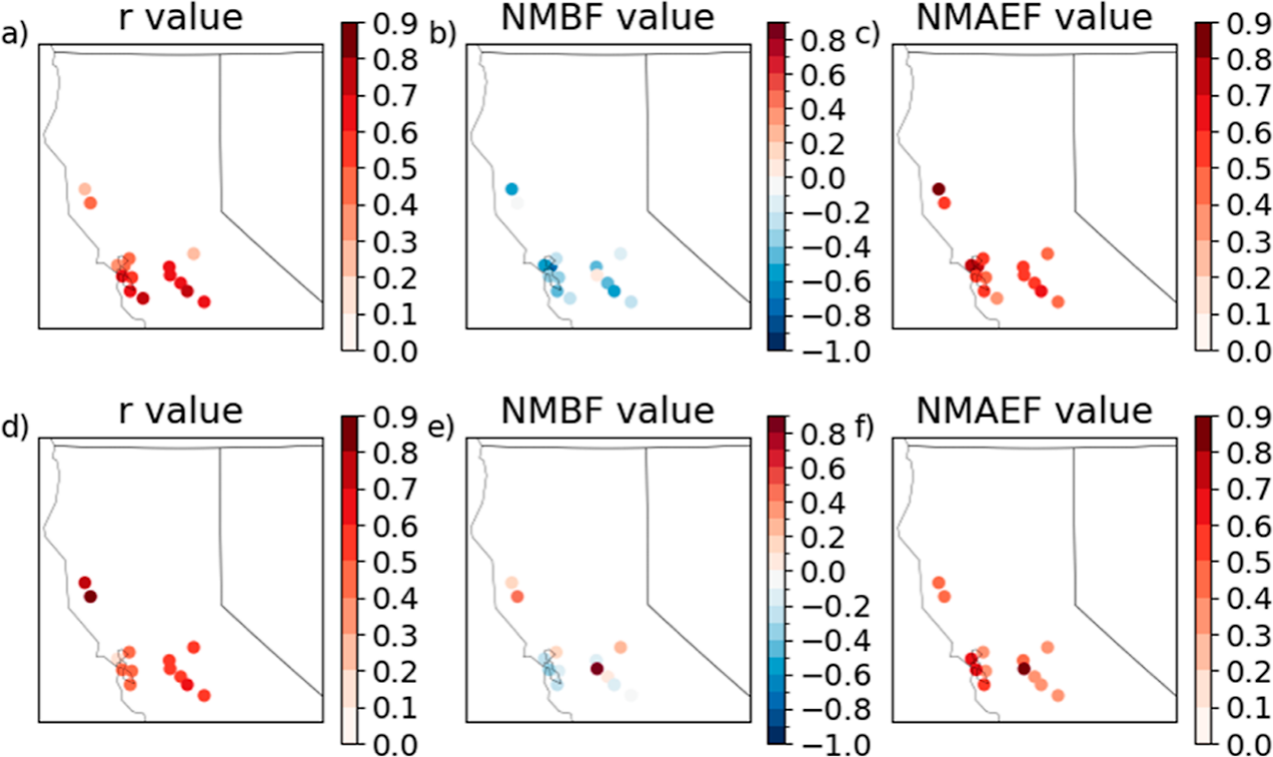
*r* value, NMBF, and NMAEF for daily modeled PM_2.5_ under the wildfire scenario and observations at each observation site averaged for the year (a–c) and averaged for June-August (d–f).

Modeled daily PM_2.5_ concentrations from the CMAQ simulation with wildfire emissions were evaluated against observations of daily average PM_2.5_ concentrations from 64 EPA stations across northern California (Figure 3). Observations were compared with the nearest neighbor grid cell to each station. Although comparing point measurements and gridded values can be problematic, particularly for grid cells that are not well mixed, it can still be helpful for assessing major biases in the model. The average *r* value was 0.55, and the NMAEF was 0.59 over the whole year. The model slightly underestimated PM_2.5_ with a normalized mean bias factor (NMBF) of −0.36. Considering only the summer months (June–August) when the impact of wildfires is strongest, the model performance improved (Figure 3, bottom panels), with an average NMAEF of 0.51 and an average NMBF of 0.06. The underestimation of PM_2.5_ by the model, particularly in nonsummer months, is likely due to an underestimation of anthropogenic emissions in the region. Given the focus on fire emissions and their impacts, we believe that these are being simulated sufficiently to support the relative analysis of wildfires and prescribed burns. Therefore, no changes were made to improve the evaluation against observations.

**Figure 4:**
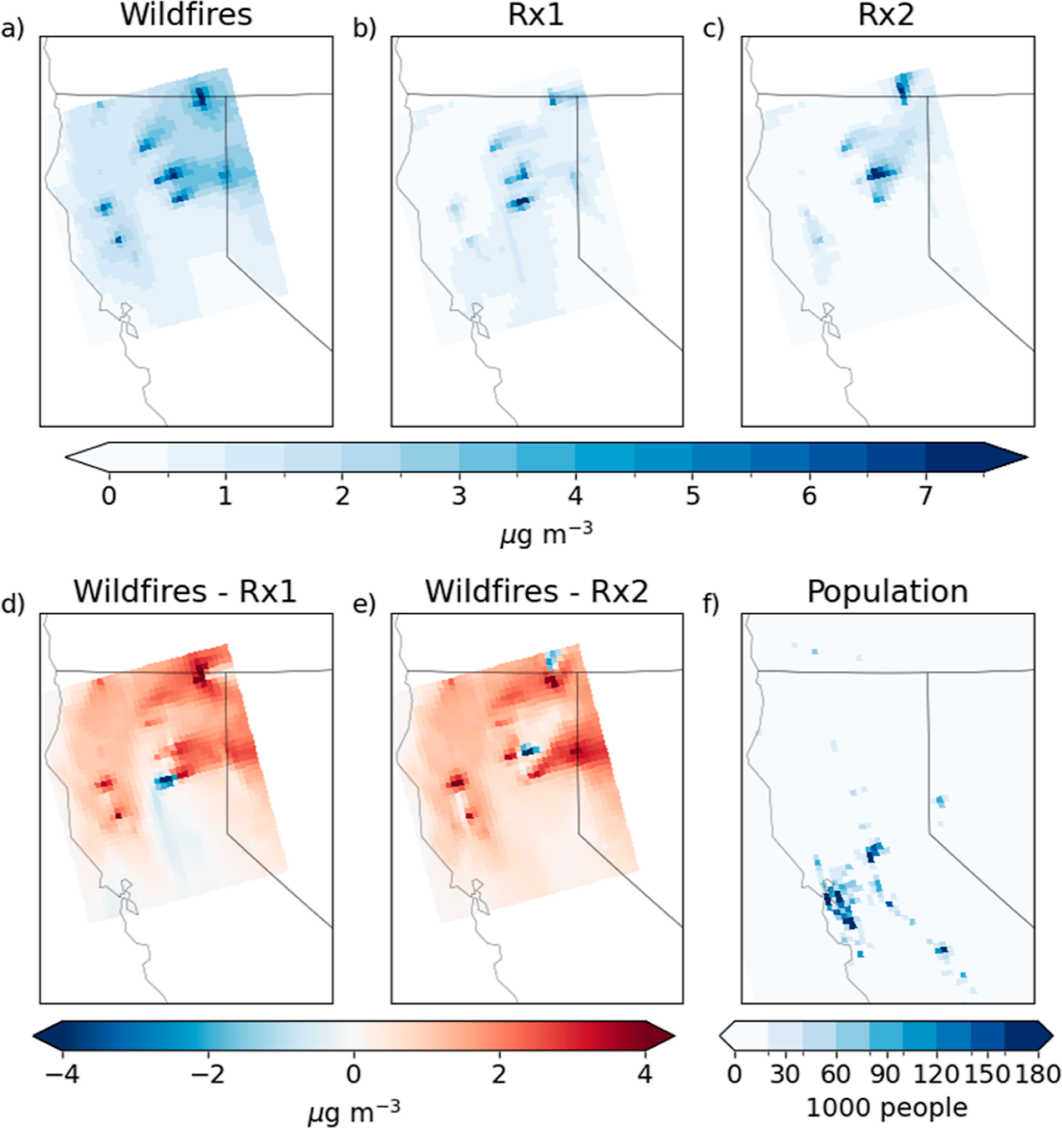
Increase in annual average PM_2.5_ mass concentration caused by fires under the wildfire (a), Rx1 (b), and Rx2 (c) scenarios. The difference in annual average PM_2.5_ concentration between the wildfire and Rx1 (d) and Rx2 (e) scenarios; red indicates concentrations greater under the wildfire scenario, and blue indicates concentrations greater under the respective prescribed burn scenarios. Gridded population in the domain (f).

**Figure 5:**
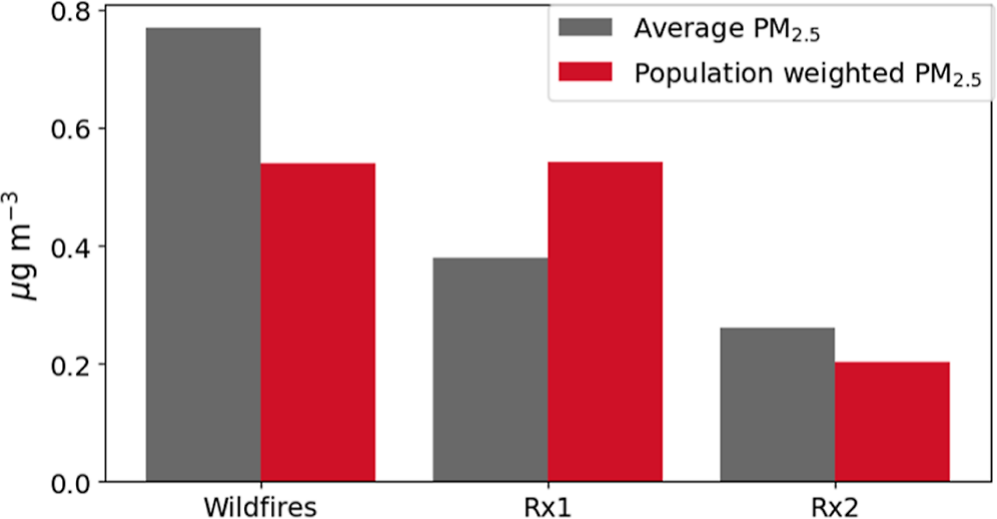
Modeled annual average surface fire-derived PM_2.5_ concentrations and population-weighted PM_2.5_ across the study area under the wildfire and Rx1 and Rx2 scenarios.

Figure 4 shows the annual average fire-derived PM_2.5_ mass concentrations for the three fire scenarios. The increase in the annual average PM_2.5_ mass concentration was 0.77 μg/m^3^ under the wildfire scenario, 0.38 μg/m^3^ under the Rx1 scenario, and 0.26 μg/m^3^ under the Rx2 scenario (Figure 5). Seasonally, summer (Jun-Aug) average PM_2.5_ concentrations increased by 2.63 μg/m^3^ under the wildfire scenario, while spring (March-May) average concentrations increased by 0.91 and 0.38 μg/m^3^ in the Rx1 and Rx2 scenarios, respectively, and fall (Sept-Nov) average concentrations increased by 0.46 and 0.66 μg/m^3^. Annual concentrations were greater under the wildfire scenario than under the Rx1 scenario for most locations, with the exception of the southern part of the study area (Figure 4). This is likely due to differing wind directions causing emissions from certain fires to move north under the wildfire scenario and south under the Rx1 scenario. When compared with the Rx2 scenario, concentrations were greater under the wildfire scenario everywhere except for a few grid cells in the center of the domain. The PM_2.5_ concentrations during the spring, summer, and fall burn periods are shown in Figure S5.

### Exposure and Health Impacts

All fire scenarios resulted in increased exposure to PM_2.5_ relative to the no-fire scenario. In the absence of fire emissions, there was no exposure to PM_2.5_ mass concentrations greater than 45 μg/m^3^, while peak exposure under each fire scenario exceeded 150 μg/m^3^, reflecting the impact that these emissions can have on local communities. The patterns of exposure between the wildfire and prescribed burn scenarios are complex and reflect differences in meteorology and population density in the study area. Under the wildfire scenario, PM_2.5_ concentrations increased across the domain. While the highest concentrations were in the northern part of the domain, a noticeable increase in PM_2.5_ occurred over populated areas in the southern part of the domain (Figure 4). Under the Rx1 scenario, PM_2.5_ concentrations increased around the fires and in the southern part of the domain, coinciding with areas of high population. Under the Rx2 scenario, PM_2.5_ concentrations increased mostly in the northern part of the domain, away from the populated areas. The population-weighted PM_2.5_ is therefore similar for the wildfire and Rx1 scenarios, despite the average fire-derived PM_2.5_ concentrations being significantly lower under the Rx1 scenario (Figure 5). Under the Rx2 scenario, the population-weighted PM_2.5_ was reduced considerably (Figure 5), reflecting the potential benefits of optimizing prescribed burn timing to minimize exposure.

Total estimated excess mortality within the study area from exposure to fire-derived PM_2.5_ was 40 deaths (39.7–40.4 with a 95% uncertainty interval) with fires under wildfire conditions, 39 deaths (39.1–39.9) under the Rx1 scenario, and 15 deaths (14.9–15.1) under the Rx2 scenario. While the PM_2.5_ emissions and average concentrations for the Rx1 and Rx2 scenarios were similar and substantially lower than for the wildfire scenario, the mortality impacts were similar for the wildfire and Rx1 scenarios and reduced for the Rx2 scenario. This is due to the higher population-weighted exposure in the Rx1 scenario, in which fire emissions were transported to the more populated southern part of the domain. Most emissions were not transported toward these highly populated areas under the wildfire scenario, resulting in a larger exposure under the Rx1 scenario than under the wildfire scenario relative to emissions. Under the Rx1 scenario, 28 of the 39 (72%) excess deaths from fires occurred during three 1–3 day periods (April 29th–30th, May 30th–June 1st, and October 26th), when only 15% of the total fire PM_2.5_ emissions were emitted. By excluding these periods in the Rx2 scenario, PM_2.5_ concentrations in highly populated areas were not as high, and population-weighted exposure was reduced. The combination of reduced emissions and favorable transport causes the mortality impact of the fires to be significantly lowered in the optimized Rx2 scenario relative to the wildfires.

## Discussion

This work shows the importance of transport when considering exposure to fire-derived PM. We considered a wildfire scenario as fires occurred and two prescribed burn scenarios, which result in high and low population-weighted exposure due to changes in transport. Much of the PM in the wildfire scenario was carried northeast away from highly populated areas, resulting in a low level of exposure. Wind direction and smoke transport in this region are variable (see Supporting Information), and if wildfires had occurred on different days, the PM could have been transported into populated areas, making the health impacts of the fires under wildfire conditions far greater. For example, Shen et al.^49^ found that wildfires in the summer of 2020 caused extreme pollution episodes across San Francisco, causing 22 excess deaths over a 42 day period. We show that even in a scenario in which smoke from prescribed fires is transported into populated areas (as in Rx1), the substantial reduction in emissions from fires burning under prescribed burn conditions compared to wildfire conditions means that the health impacts are similar to a scenario in which wildfire smoke is transported away from these populated areas. Therefore, the air quality health risk from fires under prescribed burn conditions is less than that for wildfires, using population-weighted exposure as a metric, even if meteorological conditions are unfavorable.

Of the two Rx scenarios in this study, Rx2 should be more representative of current prescribed burning practices in California since a smoke forecast is currently required for a prescribed burn to be approved and local air quality requirements must be met. The exact process for forecasting smoke, however, can vary from burn to burn. California is projected to get fewer days each year suitable for prescribed burning under a changing climate, making accurate and comprehensive risk assessment especially important for policy makers and fire practitioners working toward improved health and safety outcomes. The findings of our study emphasize the importance of including smoke forecasts and exposure impacts as part of a decision making risk assessment. We show that avoiding burn days that could lead to high exposure, something that is only possible for prescribed burns, substantially reduces the impact of fire emissions on human health.

One barrier to prescribed burning is negative public perceptions, in part from a fear of the impacts of smoke;^53^ making the public aware of the reduced air quality impacts of prescribed fires relative to wildfires could help to mitigate this barrier. The health impacts of prescribed burns may also be reduced further by prior knowledge of the risk, something which has not been modeled in our study. As prescribed burns are planned ahead of time, residents of nearby areas can be alerted to the fire beforehand and may be able to reduce their exposure by remaining indoors with doors and windows closed.^54^ We have weighted exposure equally, but some studies have shown that elderly and disadvantaged communities are more at risk from fire smoke due to prior health complaints and the inability to filter inside air.^55,56^ Burn plans could be weighted toward days which avoid increasing air pollution in these communities.

Limitations and simplifications of our study, such as in the emissions scenarios chosen, could affect our results in multiple ways. The scenarios considered in this study were chosen to allow for a direct comparison of emissions and health impacts for fires under representative wildfire and prescribed burn conditions. They do not, however, reflect a true estimate of the ability of prescribed burns to mitigate the impacts of future wildfires on air quality and human health. Since wildfire locations are determined by available fuel, ignition point, and environmental conditions, it would be impossible to set prescribed burns solely where wildfires would occur and with an identical burned area. Furthermore, prescribed burns will not entirely eradicate summer wildfires. A more realistic and achievable prescribed burn upscaling would result in a complex and climate-dependent mixture of strategically placed prescribed burns and wildfires with a much lower severity. Ideally, the effects of prescribed burn upscaling on wildfire likelihood, extent, and intensity would be modeled as a part of a comprehensive air quality analysis.^57^

Furthermore, some of the areas burned in our study are much larger than the sizes planned for prescribed fires [average fire size is 140 ha, maximum is 128,800 ha (1.4 and 1288 km^2^, respectively)]. Increasing burn sizes to encompass thousands of hectares at a time has both efficiency and ecological impact gains, but one barrier to this is the uncertainty of the air quality impacts of large prescribed fires.^58^ Our work shows that prescribed burning on the same scale as wildfires could still reduce air quality impacts relative to wildfires. It is uncertain how much area would need to be burned under prescribed burns to successfully mitigate catastrophic wildfires. If a larger area than that modeled in this study needed to burn, or if it needed to burn several times, that could negate some of the improvement to air quality. Given that one of the objectives of wildfire management is to prevent the direct impacts of fires, such as damage to infrastructure and people, prescribed burning may be considered with little to no benefit to air quality.

There are also limitations introduced by the model design. In this study, CMAQ was run offline with no feedback between the fire emissions and the meteorology. Reduced particulate emissions, as seen under the Rx scenarios, have been shown to result in increased cloud, increased rain, and a higher, less stable boundary layer.^44^ This could result in reduced surface PM_2.5_ concentrations under the Rx scenarios compared with those shown here.

Further limitations of this study are the temporal and spatial scales that are considered. Prescribed burns can reduce fuel loading for several years after the burn, meaning that a reduction in the severity of wildfires might be seen for several years. Moreover, the air quality and subsequent health impact of fires can extend far beyond the region where they occur, and there is likely to be exposure to PM from these fires outside of the region modeled here. This is particularly true under the wildfire scenario, where larger emissions of PM are more likely to lead to long-range transport.

Despite the limitations discussed, the simplified methodology used in this study contributes to the growing body of literature on wildfires and prescribed burns by more directly comparing the impacts of the two fire types. Prescribed burns and wildfires have a complex relationship which is difficult to model, and large uncertainties could mitigate the benefit of a comparison where their relationship is modeled together with the air quality impacts.

One method that has been used to compare the impacts of wildfires and prescribed burns is to calculate the health impact per unit area burned.^59^ One issue with this methodology, however, is that not all fires are equal, and an average health impact per hectare cannot necessarily be applied to other burns. This is particularly true for future fire regimes, where wildfires and prescribed burns may become common in new areas due to a changing landscape and climate.

By directly comparing the impacts of fires burning under wildfire and prescribed burn conditions, we quantitatively show that prescribed burning can have a reduced impact on adverse air quality and human health compared with wildfires. Fires burning under prescribed burn conditions have lower emissions of PM_2.5_, CO, and CO_2_ than fires burning under wildfire conditions, with emissions of PM_2.5_ almost halved. This results in reduced PM_2.5_ concentrations over much of northern California. Our results support the current regulations in California for smoke exposure to be considered before permitting prescribed burns and show that even under unfavorable transport conditions, the reduction in emissions when fires burn under prescribed burn conditions can be enough to mitigate increased exposure. The results also show that scaling up prescribed burn practices can still result in reduced health impacts.

## Supporting Information Available

Additional methodology detail for estimating fuel moisture and determining periods of high exposure and regional wind direction, CO_2_ emissions estimates, burn metrics by fuel type, and PM_2.5_ concentrations by the season (PDF)
