# Supplementary material for: California Case Study of Wildfires and Prescribed Burns: PM2.5 Emissions, Concentrations, and Implications for Human Health
Source: Environ Sci Technol. 2024 Mar 14;58(12):5210–9. doi: 10.1021/acs.est.3c06421 (PMC10976878; doi:10.1021/acs.est.3c06421)
Supplement: Supplementary file 1 — es3c06421_si_001.pdf [file es3c06421_si_001.pdf]

A California Case Study of Wildfires and Prescribed Burns: $PM_{2.5}$ Emissions, Concentrations, and Implications for Human Health

Laura Kiely, Soroush E. Neyestani, Samiha Binte-Shahid, Robert A. York, William C. Porter, Kelley C. Barsanti

Summary:
This supplementary information consists of 8 pages and contains supplementary figures S1 to S6 and supplementary tables S1 to S4.

<u>Estimating fuel moisture from soil moisture</u>

Figure S1 shows the average daily soil moisture for Northern California from the Modern-Era Retrospective analysis for Research and Applications meteorological data product (MERRA-2; GMAO, 2015) for 2012, from which the following categories were inferred: very dry (soil moisture < 0.1 m^3/m^3), dry (soil moisture between 0.1 and 0.15 m^3/m^3), moderate (soil moisture between 0.15 and 0.25 m^3/m^3), moist (soil moisture between 0.25 and 0.3 m^3/m^3), wet (soil moisture between 0.3 and 0.38 m^3/m^3), and very wet (soil moisture > 0.38 m^3/m^3). In all scenarios, the fuel moisture category used to calculate emissions was determined from the soil moisture at that location and time, and the fuel moisture values were applied as recommended in the BlueSky modelling framework for each category (see Table S1).

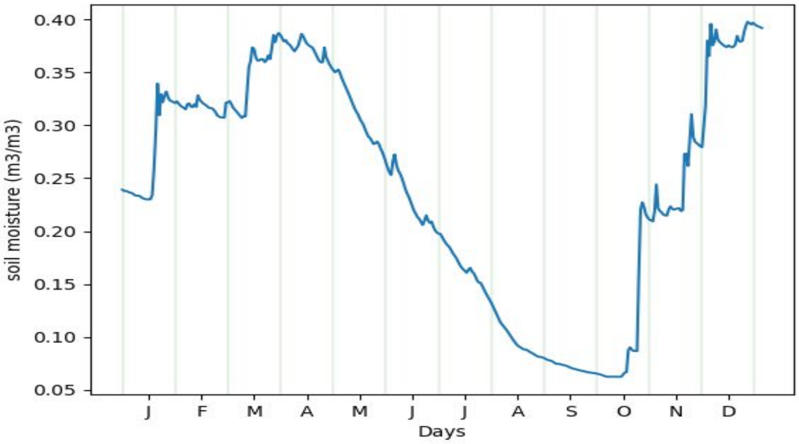

Figure S1: Average daily soil moisture content for Northern California for 2012.

Table S1: Soil and fuel moisture values for different moisture categories.

Moisture Category	Very dry	Dry	Moderate	Moist	Wet	Very Wet
Soil moisture m^3/m^3	<0.1	0.1 - 0.15	0.15 - 0.25	0.25 - 0.3	0.38 - 0.38	>0.38
1000 hr fuel moisture %	10	15	30	35	40	60
Duff fuel moisture %	20	40	75	100	130	180

Temporal and vertical distributions applied to fire emissions

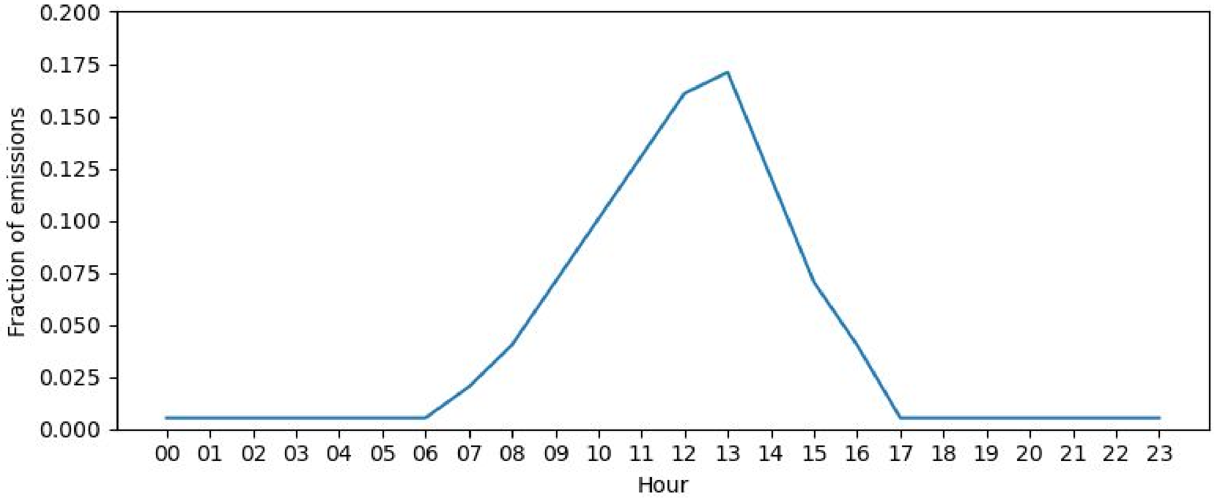

Figure S2: The temporal distribution of emissions throughout the day, as provided with the fire emissions pre-processor (Henderson, 2021).

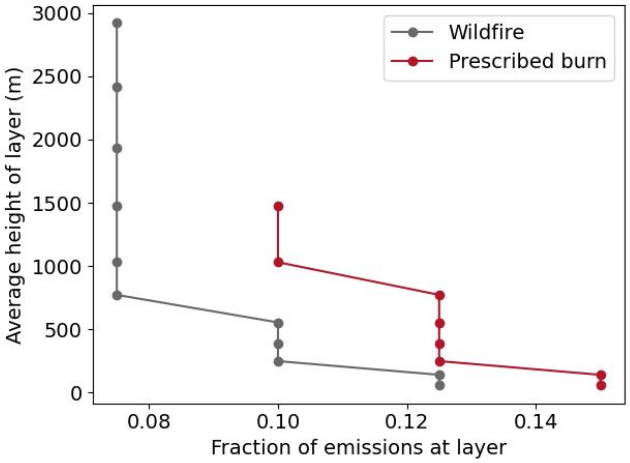

Figure S3: The vertical distribution of emissions. The average height of each layer in the model is shown on the y axis. The vertical distributions were edited from the vertical distribution provided with the fire emissions pre-processor (Henderson, 2021) using observed top heights of wildfire and prescribed burn plumes (Sofiev et al., 2013; Mallia et al., 2020).

Determining periods with high exposure

Population-weighted exposure to $PM_{2.5}$ from fires will depend on the amount of $PM_{2.5}$ emitted to and formed in the atmosphere, the transport and dilution of the emissions, and population density. Fire practitioners use transport models to forecast whether prescribed burns might cause poor air quality. We used the CMAQ simulation with the Rx1 emissions scenario to determine periods when fires caused the highest population-weighted exposure and consequently greatest

health impacts. Figure S4 shows the daily emissions and excess mortality from fires for the wildfire and Rx1 scenarios. Under the Rx1 scenario there are three periods of time (April 29th-30th, May 30th – June 1st, October 26th) when most of the excess mortalities from fires occur. These periods do not always coincide with the periods with high emissions and indicate the influence of meteorology.

Burn days which resulted in high exposure to fire-dervied $PM_{2.5}$, such as those shown in Figure S4, were therefore discounted as potential burn days for the Rx2 emissions scenario. These periods were:

- April 27th – May 2nd
- May 28th – June 2nd
- October 26th – 28th
- November 2nd – 5th

The periods of April 27th – May 2nd , May 28th – June 2nd, and October 26th – 28th were discounted first based on mortality from scenario Rx1 (Figure S4), and then, after recalculating the emissions, November 2nd – 5th was also discounted due to a similarly high mortality from fires on this day.

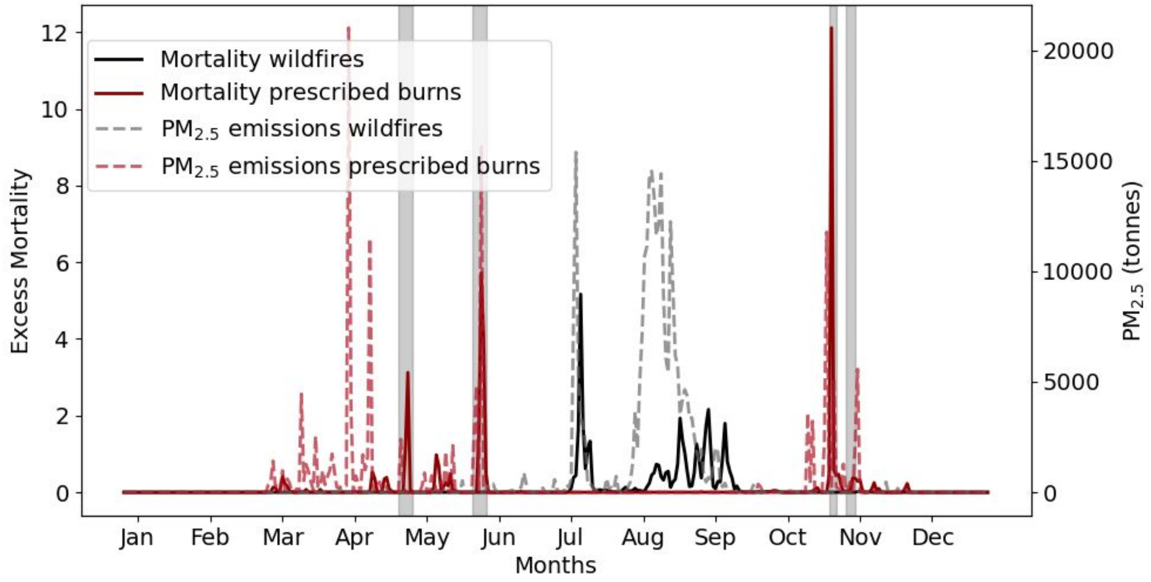

Figure S4. Excess mortality as a result of fire emissions and daily total fire emissions for both wildfire (grey) and the Rx1 (red) scenarios. The daily $PM_{2.5}$ emissions are shown on the right axis. Days which were discounted for prescribed burns in the Rx2 scenario are shaded.

Table S2. Emissions factors for CO_2 and CO in g/kg

Cover Type	CO_2	CO
Western Forest – Rx STFS*	1598	105
Western Forest – WF STFS*	1600	135
Shrubland STFS*	1674	74
Grassland STFS*	1705	61
Woody RSC**	1408	229
Duff RSC**	1371	257

* Short-term flaming and smouldering

** Residual smouldering combustion

Table S3. Emissions of CO and CO_2 under the wildfire and prescribed burn scenarios.

	Wildfires	Rx1	Rx2
CO (Tg)	1.73	0.94 (46%)	0.96 (45%)
CO_2 (Tg)	16.25	9.65 (41%)	9.76 (40%)

Table S4. Burned area, fuel loading and fuel consumption of the fires under the wildfire and prescribed burn scenarios.

	Wildfire			Rx1			Rx2		
	Trees	Shrub	Grass	Trees	Shrub	Grass	Trees	Shrub	Grass
Burned area (km^2)	4450	3592	1837	4450	3592	1837	4450	3592	1837
Fuel loading (Tg)	16.40	4.16	0.93	16.40	4.16	0.93	16.40	4.16	0.93
Fuel consumption (Tg)	9.45	1.00	0.11	5.22	0.98	0.11	5.30	0.98	0.11

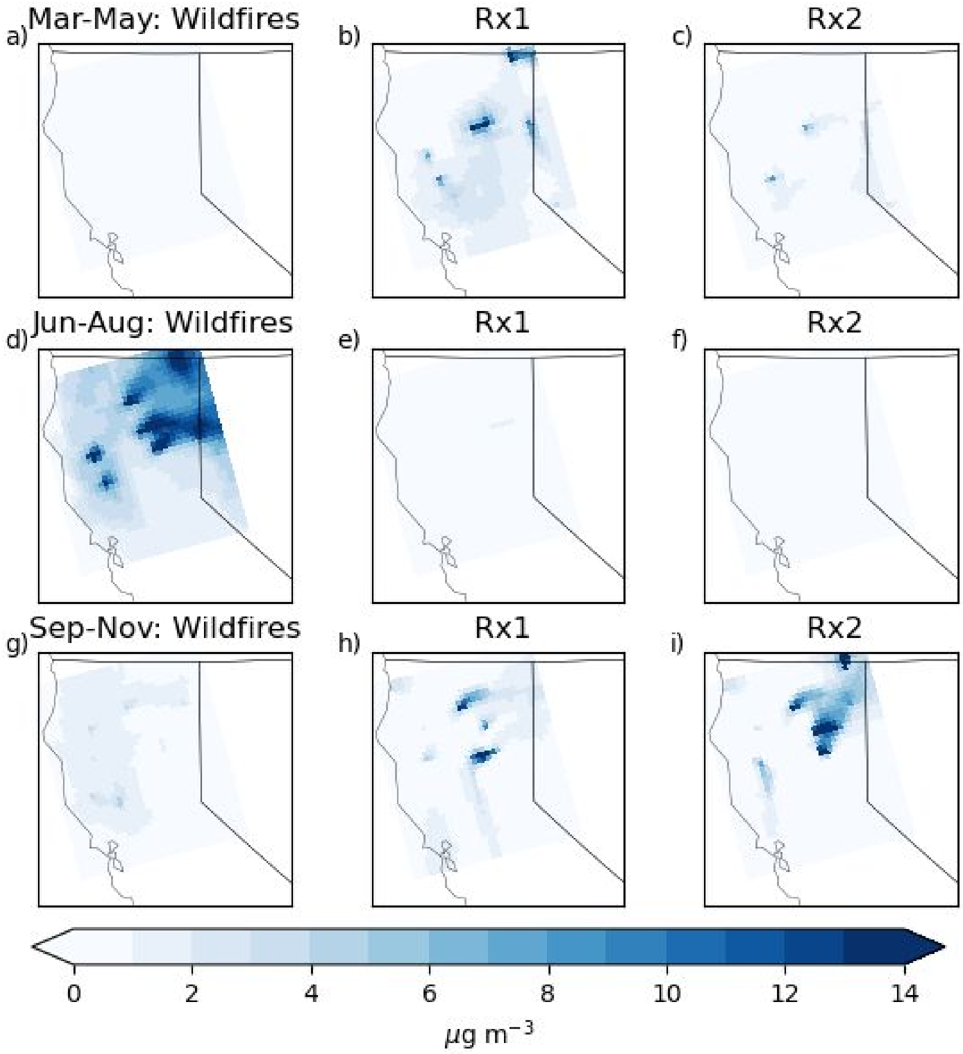

Figure S5: Modelled three-month seasonal averages, March -May (a-c), June-August (d-f) and September-November (g-i), of the increase in $PM_{2.5}$ from fires for wildfire and prescribed burn scenarios relative to the no fire scenario. December to February had minimal fire emissions and is not shown.

<u>Regional wind direction</u>

Figure S6 shows the variability in the wind direction within each month in the study area over a 5 year period, including the 2012 study year. Generally, there are more south and east winds (red and yellow tones) than north and west (blue and green tones) in the region, particularly in summer. However, there are winds in all directions in every month, and the distribution of wind directions changes between years, suggesting there is no dominant transport pattern in the region.

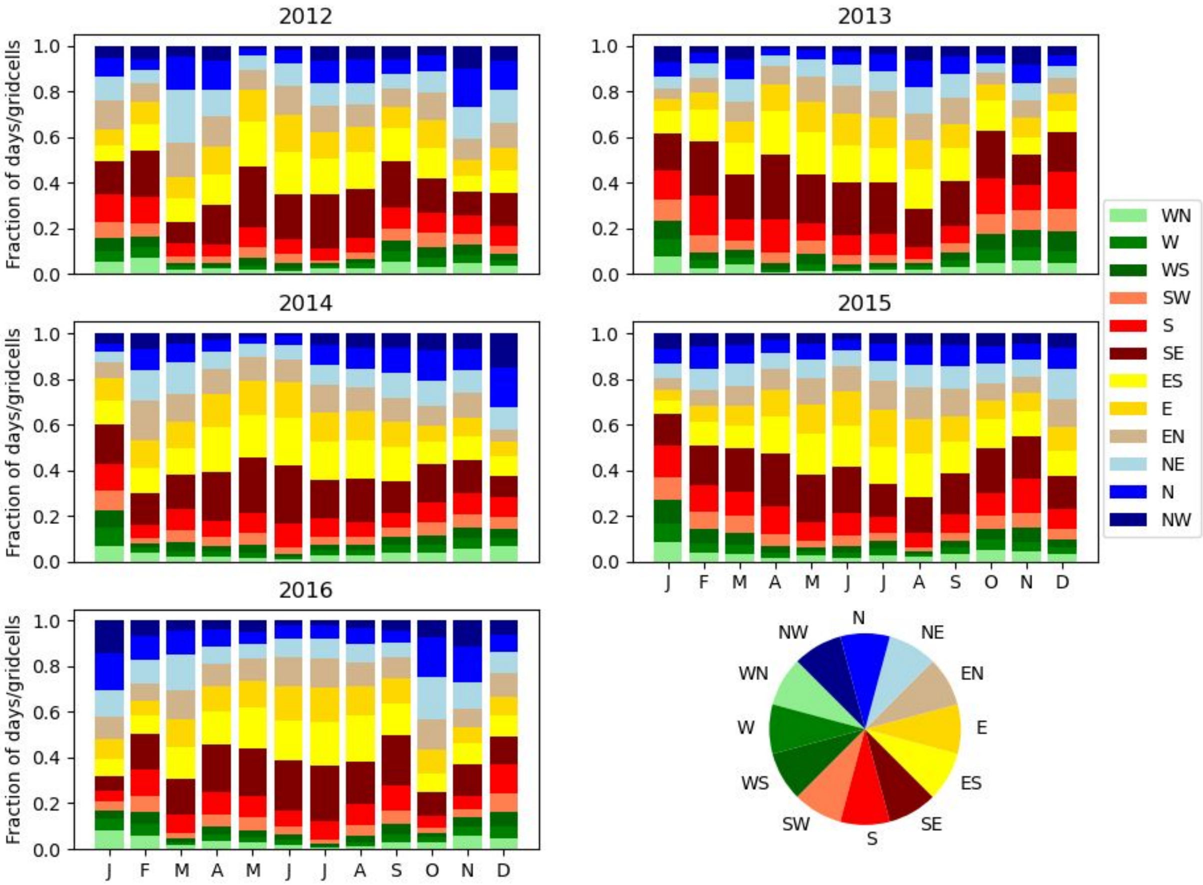

Figure S6: The fraction of gridcells and days in each month when the wind direction is northerly (blue tones, North-East (NE), North (N), and north-West (NW)), easterly (yellow tones, East-South (ES), East (E), East-North (EN)), southerly (red tones, South-West (SW), South (S), South-East (SE)), and westerly (green tones, West-North (WN), West (W), West-South (WS)).
